# *Toxoplasma* Effector TgIST Targets Host IDO1 to Antagonize the IFN-γ-Induced Anti-parasitic Response in Human Cells

**DOI:** 10.3389/fimmu.2018.02073

**Published:** 2018-09-19

**Authors:** Hironori Bando, Naoya Sakaguchi, Youngae Lee, Ariel Pradipta, Ji Su Ma, Shun Tanaka, De-Hua Lai, Jianfa Liu, Zhao-Rong Lun, Yoshifumi Nishikawa, Miwa Sasai, Masahiro Yamamoto

**Affiliations:** ^1^Department of Immunoparasitology, Research Institute for Microbial Diseases, Osaka University, Osaka, Japan; ^2^Laboratory of Immunoparasitology, WPI Immunology Frontier Research Center, Osaka University, Osaka, Japan; ^3^State Key Laboratory of Biocontrol, Center for Parasitic Organisms, School of Life Sciences, Guangzhou, China; ^4^Department of Pathology and Pathogenic Biology, Medical College of Ningbo University, Ningbo, China; ^5^National Research Center for Protozoan Diseases, Obihiro University of Agriculture and Veterinary Medicine, Obihiro, Japan

**Keywords:** IFN-γ, IDO1, IDO2, virulence, human, TgIST

## Abstract

*Toxoplasma gondii* is an important human and animal pathogen that causes life-threatening toxoplasmosis. Interferon-γ (IFN-γ) is critical for anti-*T. gondii* cell-autonomous immunity in both humans and mice. To proliferate efficiently within the hosts, virulent strains of *T. gondii* can suppress IFN-γ-dependent immunity. During parasite infection, it is well-characterized that various virulence effectors are secreted to transcriptionally or post-translationally target IFN-γ-inducible GTPases, which are essential for anti-parasite responses in mice. However, the role of IFN-γ-inducible GTPases in anti-*T. gondii* responses in human cells is controversial since they are non-functional or absent in humans. Instead, IFN-γ-induced tryptophan degradation by indole-2,3-dioxygenase (IDO) is important for the anti-*T. gondii* human response. To date, the *T. gondii* virulent mechanism targeting IDO in human cells remains elusive. Here we show that although humans possess two IDO isozymes, IDO1 and IDO2, human cells of various origins require IDO1 but not IDO2 for IFN-γ-induced cell-autonomous immunity to *T. gondii. T. gondii* secretes an effector TgIST to inhibit IDO1 mRNA expression. Taken together, the data suggests that *T. gondii* possesses virulence programs operated by TgIST to antagonize IFN-γ-induced IDO1-mediated anti-parasite cell-autonomous immunity in human cells.

## Introduction

*Toxoplasma gondii* is an intracellular apicomplexan protozoan that has a broad range of intermediate hosts, including humans (1, 2). Although it is estimated that at least one-third of the world's population is infected with *T. gondii*, most of these infections are asymptomatic. However, the parasite remains in a latent state and may reactivate and lead to severe diseases including hepatitis, encephalitis, and myocarditis if that individual becomes immunocompromised (3, 4). Moreover, toxoplasmosis caused by *T. gondii* infection may lead to congenital diseases in fetuses and newborn infants from primarily-infected pregnant women (5). Thus, *T. gondii* is one of the most important human and animal pathogens.

The host immune system plays a critical role in the course of *T. gondii* infection and in the progression of toxoplasmosis. In particular, the type I cytokine interferon-γ (IFN-γ), which is produced by CD4^+^ T cells and natural killer cells (NK), is an essential host factor for anti-*T. gondii* responses in host cells (6). This is because IFN-γ activates the transcription factor STAT1 and induces the expression of hundreds of genes (7). In the mouse model, IFN-γ-induced anti-*T. gondii* responses have been extensively analyzed. Parasitocidal and parasitostatic effects mediated by IFN-γ-inducible gene products have been observed in mice. The parasitocidal effects are coordinated by IFN-γ-inducible GTPases such as p47 immunity-related GTPases (IRGs) and p65 guanylate-binding proteins (GBPs) (8, 9). These GTPases accumulate on parastitophorous vacuoles (PVs), leading to their destruction (10). In mice, the accumulation of IRGs and GBPs on *T. gondii* requires some essential autophagy-related (Atg) proteins such as Atg3, Atg5, Atg7, Atg16L1, and GABARAPs but not other Atg proteins such as Atg9, Atg14, FIP200, and LC3s (11), suggesting the non-autophagic role of these Atg proteins in IFN-γ-mediated anti-*T. gondii* responses in mice. Atg16L1-deficient murine cells are severely defective in the IFN-γ-induced clearance of *T. gondii* due to impaired recruitment of GBPs and IRGs to *T. gondii* (12, 13), suggesting the essential role of Atg16L1 in anti-*T. gondii* responses in mice. In addition, this parasitostatic mechanism involves nitric oxide (NO), which is produced by IFN-γ-inducible NO synthase (iNOS) (14). Mice lacking IRGs, GBPs, and iNOS are susceptible to *T. gondii* infection (8, 15–20). Thus, the significance of these IFN-γ-inducible factors for anti-*T. gondii* immune responses in mice has previously been established.

However, the importance of IFN-γ-inducible GTPase- and NO-mediated mechanisms in humans is less certain. For example, compared with more than 20 IRG members in mice, humans only possess one IRG, which is not inducible by IFN-γ (21). Furthermore, inhibition of NO production does not affect *T. gondii* growth in IFN-γ-stimulated human macrophages (22). Regarding GBPs, a human reprogrammed fibroblast-like cell line (HAP1) lacking all GBPs shows a normal IFN-γ-dependent reduction in *T. gondii* growth (12, 23). However, knockout of GBP1 in a human lung epithelial cell line (A549) and knockdown of GBP1 in human mesenchymal stem cells (MSCs) results in impaired restriction of *T. gondii* growth in response to IFN-γ (24, 25). Thus, the involvement of IFN-γ-inducible GTPases and NO in the human anti-*T. gondii* response is controversial (12, 23–26). Regarding the role of autophagy proteins in human cells, ATG16L1 is dispensable for IFN-γ-induced inhibition of *T. gondii* growth in HAP1 cells and HUVECs (12, 27), whereas ATG16L1 is required for anti-parasite responses in HeLa cells via IFN-γ-inducible ubiquitination of *T. gondii* PVs (23). Thus, the anti-*T. gondii* role of ATG16L1 in humans may be cell-type specific. By contrast, IFN-γ-dependent nutrient deprivation or cell death has been established as an anti-*T. gondii* response in human cells (28, 29). Regarding nutrient deprivation, IFN-γ stimulates the expression of indoleamine 2,3-dioxygenases (IDO) to degrade tryptophan, which is an essential amino acid for *T. gondii* intracellular growth (30, 31). The treatment of IFN-γ-activated human cells with a pharmacological inhibitor of IDO called 1-methyl-DL- tryptophan (1-DL-MT) leads to defects in the IFN-γ-induced reduction of *T. gondii* numbers (32), establishing the significance of IDO in the IFN-γ-induced anti-*T. gondii* response in human cells. IDO consists of two closely related family members, IDO1 and IDO2 (33). Previous studies using 1-DL-MT concluded that IDO is responsible for the IFN-γ-inducible anti-*T. gondii* response (32, 34). However, given that both IDO1 and IDO2 are sensitive to 1-DL-MT (35, 36), it remains unclear whether either IDO1 or IDO2 (or both) is more important.

To antagonize the IFN-γ-induced anti-parasitic host response, *T. gondii* secretes various effector molecules into host cells upon infection (37, 38). The effector mechanisms are also extensively analyzed in the mouse model. ROP5, ROP17, and ROP18 are secreted from the rhoptry organelles to suppress IRG/GBP-dependent immune responses at PV membranes, resulting in increased virulence in mice (39–43). In addition, a dense granule-derived effector GRA7 is also injected into host cells to enhance ROP18-mediated inhibition of IRGs in mice (44). Furthermore, *T. gondii* infection is shown to impede STAT1-mediated gene expression (45, 46). TgIST was recently shown to be secreted from dense granules and finally localized at the host nucleus, where TgIST associates with the remodeled host nucleosome and the deacetylase complex to inhibit the expression of STAT1-dependent genes including iNOS, chemokines, IRGs, and GBPs, leading to enhanced virulence in mice (47, 48). Although ROP5 and ROP18 are virulence factors in mice, these effectors do not affect the ability of *T. gondii* to survive in IFN-γ-stimulated human fibroblasts (49). Regarding TgIST, although TgIST-deficient parasites are defective in STAT1-depdendent gene expression in human cells (47, 48), whether TgIST affects parasite survival in IFN-γ-stimulated host cells and which, if any, of the STAT1-regulated human gene products is targeted by TgIST remains elusive, given the differences between humans and mice in terms of IFN-γ-induced anti-*T. gondii* cell-autonomous immunity.

In the present study, we first demonstrated that IDO1, but not IDO2, is required for IFN-γ-induced inhibition of *T. gondii* growth of human cell lines of various origins. In terms of *T. gondii* virulence mechanisms, we have shown that TgIST directly suppresses IDO1 gene expression to promote parasite growth in IFN-γ-activated human cells. Taken together, these data have revealed that *T. gondii* uses TgIST as a virulence mechanism to impede the IDO1-dependent cell-autonomous response in IFN-γ-activated human cells.

## Results

### ATG16L1-independent IFN-γ-induced reduction of *T. gondii* numbers in the human HAP1 cell line

**Figure 1:**
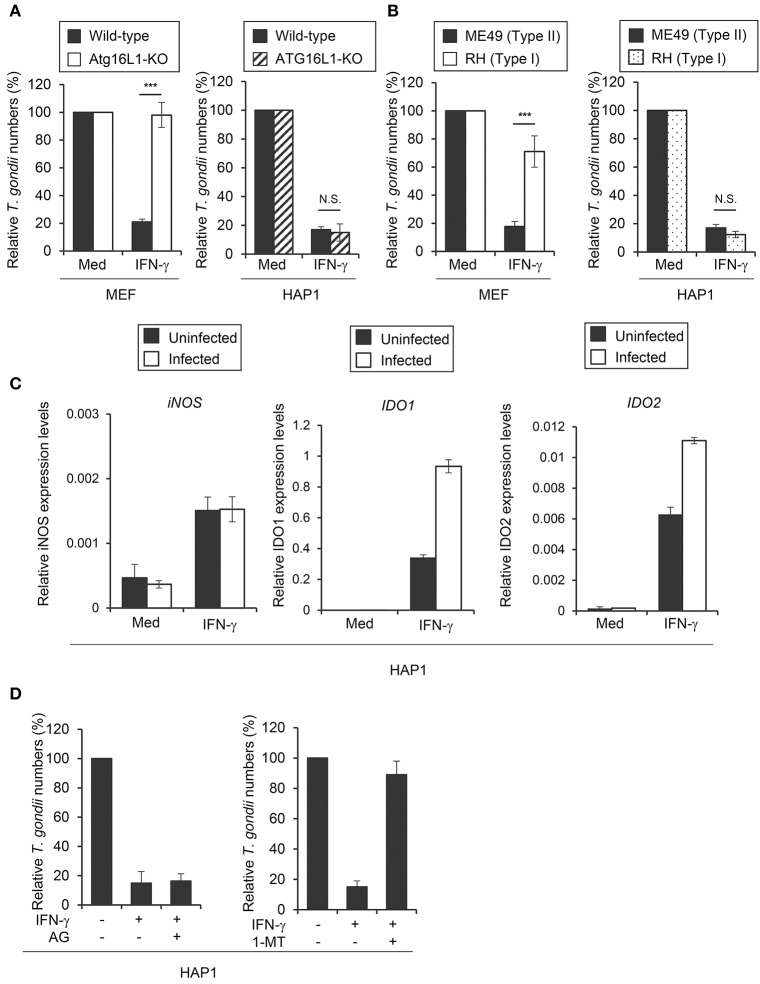
IDO plays a critical role in anti *T. gondii* response in HAP1 cells. **(A)** WT or Atg16L1-KO MEFs or HAP1 cells were untreated or pre-treated with IFN-γ for 24 h, and then infected with *T. gondii*. The parasite survival rate after 24 h post infection was measured by luciferase assay. **(B)** MEFs or HAP1 cells were untreated or pre-treated with IFN-γ for 24 h, and then infected with Type I or Type II *T. gondii*. The parasite survival rate after 24 h post infection was measured by luciferase assay. **(C)** Quantitative RT-PCR analysis of iNOS, IDO1 or IDO2 mRNA level in HAP1 cells that were untreated or treated with IFN-γ for 24 h, and then infected with or without *T. gondii* was performed. (**D**) HAP1 cells were untreated or treated with IFN-γ and/or Aminoguanidine and/or 1-DL-MT for 24 h, and then infected with *T. gondii*. The parasite survival rate after 24 h post infection was measured by luciferase assay. Indicated values are means of ± s.d. (three biological replicates per group from three independent experiments) **(A–D)**. ***p < 0.001; N.S., not significant; (Student's *t*-test).

We have previously shown that ATG16L1 plays an important role in the IFN-γ-induced reduction of type II *T. gondii* (ME49) in mouse cells (mouse embryonic fibroblasts; MEFs) but not in human HAP1 cells (Figure 1A), suggesting an ATG16L1-independent IFN-γ-induced anti-*T. gondii* response in human cells (12). To elucidate the molecular mechanism, we next challenged MEFs and HAP1 cells with type I (RH) or type II (ME49) parasites. As shown previously (12), MEFs showed more efficient IFN-γ-induced reduction of type II parasites than of type I parasites. By contrast, IFN-γ stimulation could similarly and efficiently reduce the numbers of both type I and II parasites in HAP1 cells (Figure 1B). IFN-γ-induced degradation of arginine by iNOS, or of tryptophan by IDO, which consists of two members, IDO1 and IDO2, have been shown to be important for the anti-*T. gondii* response in mouse or human cells (18, 30, 31). To test whether iNOS or IDO (or both) are involved in the process in HAP1 cells, we examined iNOS, IDO1, or IDO2 mRNA expression in HAP1 cells (Figure 1C). Stimulation of IFN-γ led to strong induction of IDO1 and IDO2 mRNAs and weak induction of iNOS mRNA. Second, we tested the expression levels of these mRNAs in IFN-γ-stimulated cells followed by *T. gondii* infection (Figure 1C). *T. gondii* infection at 24 hours after IFN-γ stimulation did not interfere with the expression of iNOS, IDO1, and IDO2 mRNAs in HAP1 cells (Figure 1C). Furthermore, we treated HAP1 cells with a pharmacological inhibitor of iNOS known as aminoguanidine or an inhibitor of IDO known as 1-methyl-DL-tryptophan (1-DL-MT), and compared the parasite numbers. 1-DL-MT but not aminoguanidine treatment abolished the IFN-γ-induced reduction of *T. gondii* numbers in HAP1 cells (Figure 1D), strongly suggesting the anti-*T. gondii* function of IDO in the human HAP1 cell line.

### IDO1 but not IDO2 plays a critical role in the anti-*T. gondii* response in HAP1 cells

**Figure 2:**
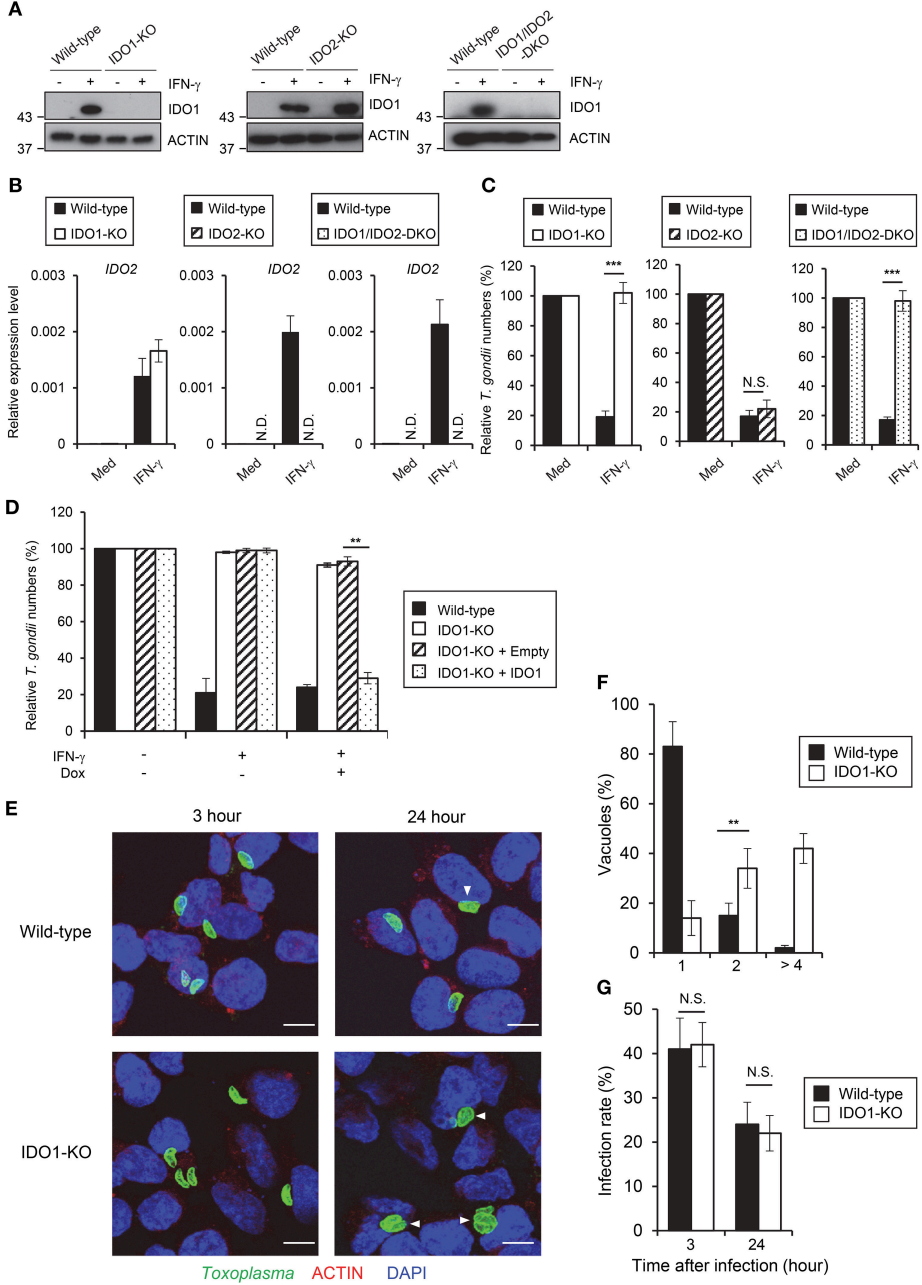
IDO1 but not IDO2 has a critical role in anti-*T. gondii* response in HAP1 cells. **(A)** WT, IDO1-KO, IDO2-KO, or IDO1/IDO2-DKO HAP1 cells were stimulated with IFN-γ for 24 h, and then lysates were detected by Western blot. **(B)** Quantitative RT-PCR analysis of IDO2 mRNA level in IDO1-KO, IDO2-KO, or IDO1/IDO2-DKO HAP1 cells that were untreated or treated with IFN-γ for 24 h was performed. **(C)** WT, IDO1-KO, IDO2-KO, or IDO1/IDO2-DKO HAP1 cells were untreated or treated with IFN-γ for 24 h, and then infected with *T. gondii*. The parasite survival rate after 24 h post infection was measured by luciferase assay. **(D)** WT, IDO1-KO, IDO1-KO+empty, or IDO1-KO+IDO1 HAP1 cells were untreated or treated with IFN-γ for 24 h, and then infected with *T. gondii*. The parasite survival rate after 24 h post infection was measured by luciferase assay. **(E)** Fluorescence confocal microscopy of WT and IDO1-KO HAP1 cells stimulated by IFN-γ for 24 h, subsequently infected with *T. gondii* for 3 or 24 h, and immunostained for ACTIN (red) and *T. gondii* GAP45 (green). The nucleus was stained with DAPI (blue). Arrow heads show vacuoles containing 2 or more parasites. Scale bars correspond to 5 μm. **(F,G)** WT or IDO1-KO HAP1 cells were stimulated with IFN-γ for 24 h, and then infected with *T. gondii*. The parasite number per vacuole after 24 h post infection **(F)** or the parasite infection rate after 3 or 24 h post infection **(G)** was measured by IFA. Western blot and immunofluorescence images are representative of three independent experiments **(A,E)**. Indicated values are means of ± s.d. (three biological replicates per group from three independent experiments) **(B,C,D,F,G)**. ***p < 0.001, **p < 0.01; N.S., not significant; (Student's *t*-test). N.D., not detected.

Both IDO1 and IDO2 could be inhibited by 1-DL-MT (35, 36). Although both IDO1 and IDO2 mRNAs were highly induced by IFN-γ stimulation in HAP1 cells (Figure 1C), it remained to be seen which was more important for the IFN-γ-induced response in HAP1 cells. To clarify the contribution of IDOs in HAP1 cells, we generated IDO1 singly deficient (IDO1-KO), IDO2 singly deficient (IDO2-KO), and doubly deficient (IDO1/IDO2-DKO) HAP1 cells by CRISPR/Cas9 genome editing (Figures 2A,B, Figure S1A) and tested the IFN-γ-induced reduction of type II parasite numbers. Although IDO2-KO HAP1 cells functioned normally, IDO1-KO cells as well as IDO1/IDO2-DKO cells were completely defective in IFN-γ-induced parasite reduction (Figure 2C), suggesting that IDO1 but not IDO2 is essential for the IFN-γ-induced anti-*T. gondii* response in the human cell line. Kynurenine is a tryptophan metabolite of IDOs (50). Therefore, we measured kynurenine concentrations in HAP1 cells lacking IDOs (Figure S1B). Whereas, the kynurenine concentrations were increased upon IFN-γ treatment in wild-type HAP1 cells, such an increment was not observed in IDO1-KO or IDO1/IDO2-DKO HAP1 cells (Figure S1B). By contrast, IDO2-KO cells showed normal induction of kynurenine after IFN-γ stimulation (Figure S1B), correlating with the importance of IDO1 in the IFN-γ-induced reduction of parasite numbers and the degree of tryptophan degradation. In mouse cells, a *T. gondii* strain-dependent difference was observed in the IFN-γ-induced anti-*T. gondii* response (Figure 1B) (12, 13). By contrast, although wild-type cells exhibited greatly reduced numbers of type I parasites after IFN-γ stimulation, this IFN-γ-mediated reduction was not observed in IDO1-KO HAP1 cells (Figure S1C), suggesting the lack of strain dependence in this human cell line. Next, we analyzed whether IDO1 expression could rescue the defective anti-*T. gondii* response of IDO1-KO cells. To achieve this, IDO1-KO cells were transfected with a doxycycline-inducible IDO1 or the empty control vectors and the IFN-γ-induced anti-*T. gondii* response was tested (Figure 2D, Figure S1D). The doxycycline-inducible IDO1 expression led to a reduction of parasite numbers in the IFN-γ-stimulated IDO1-KO cells (Figure 2D). Next we tested whether IDO1 plays a role in the inhibition of *T. gondii* replication or in parasite elimination. The parasite numbers per vacuole in IFN-γ-stimulated IDO1-KO HAP1 cells were significantly higher than those of wild-type cells (Figures 2E,F). Whereas, the rates of *T. gondii*-infected wild-type or IDO1-KO cells were comparable 3 and 24 h post infection (Figures 2E,G), indicating that IDO1 inhibits *T. gondii* replication in IFN-γ-stimulated HAP1 cells. Taken together, these data suggest that IDO1 plays a critical role in the IFN-γ-induced anti-*T. gondii* response in HAP1 cells.

### IDO1 is required for the anti-*T. gondii* response in various human cell lines

**Figure 3:**
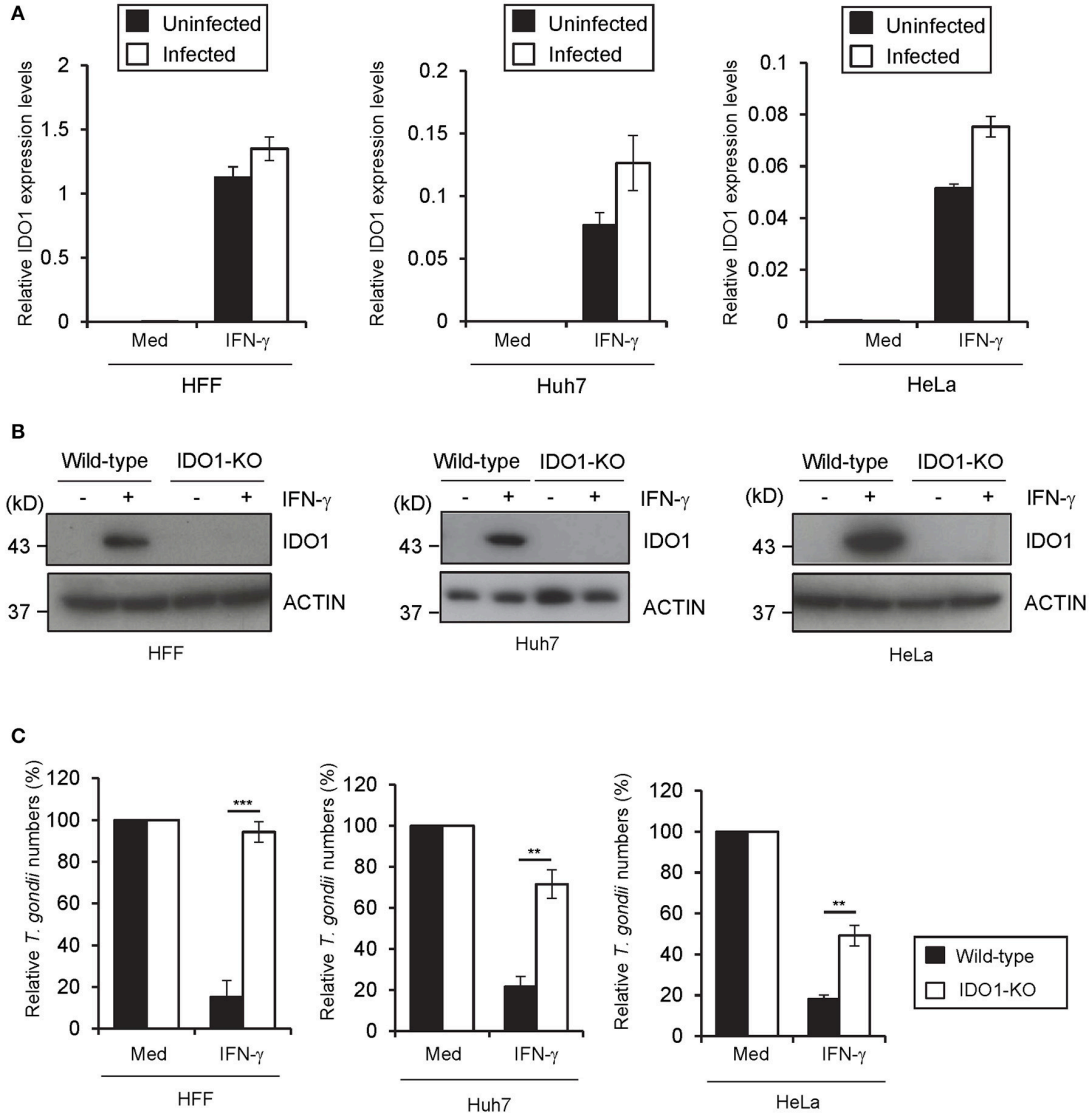
IDO1 has a critical role in anti-*T. gondii* response in various human cells. **(A)** Quantitative RT-PCR analysis of IDO1 mRNA level in HFFs, Huh7 or HeLa cells that were untreated or treated with IFN-γ for 24 h, and then infected with or without *T. gondii* was performed. **(B)** WT or IDO1-KO HFFs, Huh7 or Hela cells were untreated or treated with IFN-γ for 24 h, and then lysates were detected by Western blot. **(C)** WT or IDO1-KO HFFs, Huh7 or Hela cells were untreated or treated with IFN-γ for 24 h, and then infected with *T. gondii*. The parasite survival rate after 24 h post infection was measured by luciferase assay. Western blot image is representative of three independent experiments **(B)**. Indicated values are means of ± s.d. (three biological replicates per group from three independent experiments) **(A,C)**. ***p < 0.001, **p < 0.01; N.S., not significant; (Student's *t*-test). N.D., not detected.

Next we assessed whether IDO1 is important for the IFN-γ-induced anti-*T. gondii* response in other human cells. IDO1 mRNAs were highly induced in foreskin fibroblasts (HFFs), a hepatocyte cell line (Huh7), and an epithelial cell line (HeLa) upon IFN-γ stimulation regardless of the subsequent *T. gondii* infection (Figure 3A). Then we generated IDO1-KO HFFs, Huh7, or HeLa cells by CRISPR/Cas9-genome editing (Figure 3B, Figure S2A) and analyzed the IFN-γ-induced reduction in *T. gondii* numbers. Among all of the cell types tested, IDO1-KO cells were defective in the IFN-γ-mediated anti-*T. gondii* response (Figure 3C). However, compared with IDO1-KO HAP1 cells or HFFs, both of which displayed complete loss of an anti-parasite response (Figures 2C, 3C), IDO1 deficiency in Huh7 or HeLa cells resulted in severely impaired or modest defects (Figure 3C), suggesting an IDO1-independent anti-*T. gondii* response in Huh7 and HeLa cells. In addition, Tryptophan 2,3-dioxygenase (TDO) was not involved to IFN-γ induced anti- *T. gondii* responses in Huh7 cells (Figure S2B).

### ATG16L1 is required for the anti-*T. gondii* response in a cell type-specific manner

**Figure 4:**
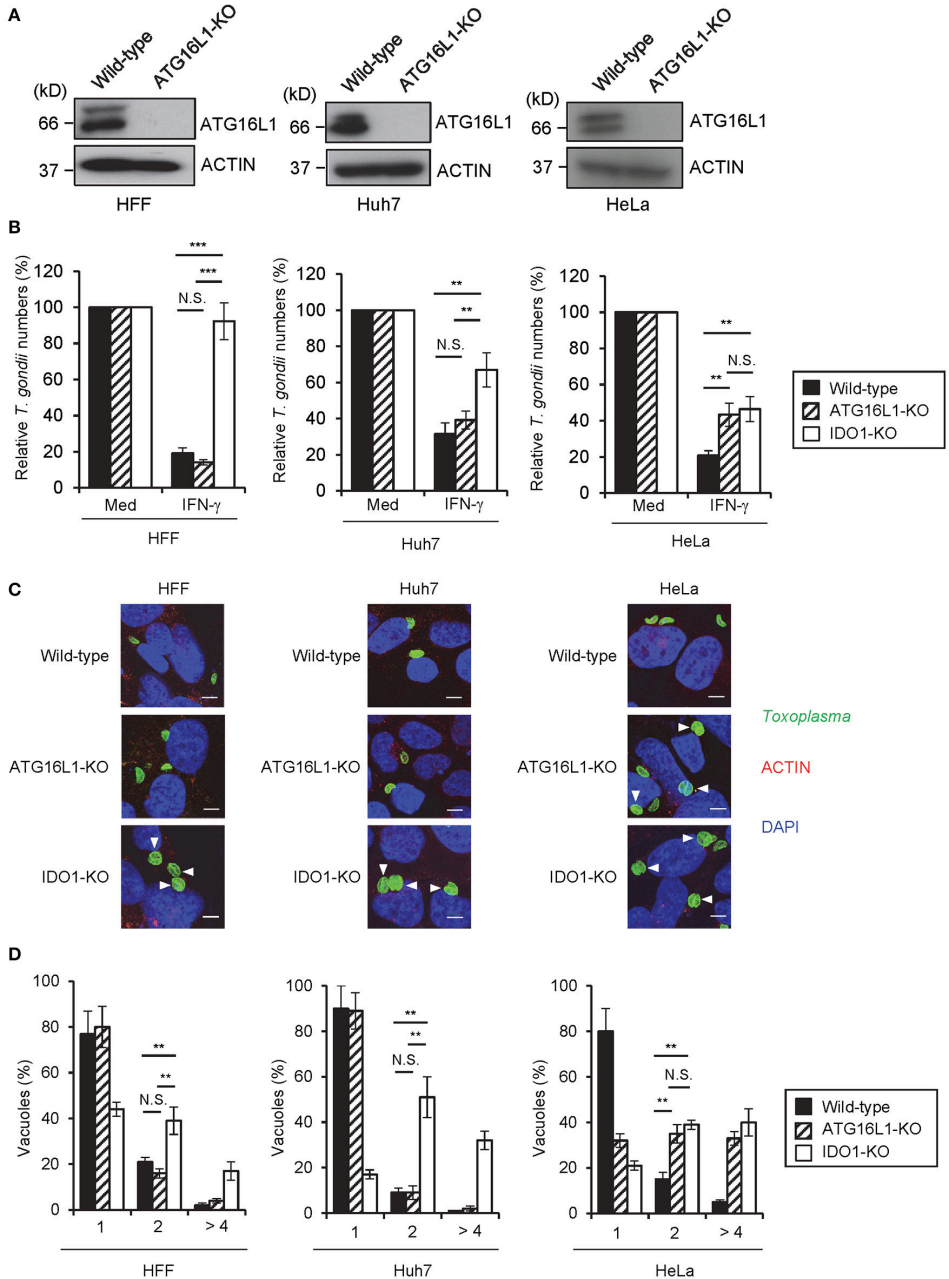
IDO1 but not ATG16L1 extensively participate in anti-*T. gondii* response in human cell lines. **(A)** WT or ATG16L1-KO HFFs, Huh7 or Hela cells were untreated or treated with IFN-γ for 24 h, and then lysates were detected by Western blot. **(B)** WT, IDO1-KO, or ATG16L1-KO HFFs, Huh7 or Hela cells were untreated or treated with IFN-γ for 24 h, and then infected with *T. gondii*. The parasite survival rate after 24 h post infection was measured by luciferase assay. **(C)** Fluorescence confocal microscopy of WT, ATG16L1-KO and IDO1-KO HFF, Huh7 or HeLa cells stimulated by IFN-γ for 24 h, subsequently infected with *T. gondii* for 24 h, and immunostained for ACTIN (red) and *T. gondii* GAP45 (green). The nucleus was stained with DAPI (blue). Arrow heads show vacuoles containing 2 or more parasites. Scale bars correspond to 5 μm. **(D)** WT, IDO1-KO or ATG16L1-KO HFFs, Huh7 or Hela cells were untreated or treated with IFN-γ for 24 h, and then infected with *T. gondii*. The number of parasites per vacuole after 24 h post infection was measured by IFA. Western blot and immunofluorescence images representative of three independent experiments **(A,C)**. Indicated values are means of ± s.d. (three biological replicates per group from three independent experiments) **(B,D)**. ***p < 0.001, **p < 0.01; N.S., not significant; (Student's *t*-test). N.D., not detected.

A previous study demonstrated that ATG16L1 is involved in the IFN-γ-induced anti-*T. gondii* response in HeLa cells (23). Therefore, we hypothesized that ATG16L1 plays a role in the IDO1-independent response in Huh7 and HeLa cells. To examine this possibility, we generated ATG16L1-deficient (ATG16L1-KO) HFFs, Huh7, and HeLa cells by CRISPR/Cas9 genome editing (Figure 4A). Although ATG16L1-KO HFFs and Huh7 cells showed no increment of parasite numbers (Figure 4B) or parasite numbers per vacuole upon IFN-γ stimulation (Figures 4C,D), ATG16L1-KO HeLa cells exhibited modest defects (Figures 4C,D). Furthermore, 1-DL-MT treatment in ATG16L1-KO HeLa cells resulted in a less efficient IFN-γ-mediated response than in non-treated control cells (Figure S2C), suggesting that ATG16L1 is involved in the IFN-γ-induced anti-parasite response in a cell type-specific manner. Thus, IDO1 is involved in the IFN-γ-induced anti-*T. gondii* response in human cells of various origins (**Figure 6A**). However, given the dispensable role of IDO in HUVECs (51), the degree of importance of IDO1 in the anti-*T. gondii* response depends on cell type.

### TgIST directly suppresses IDO1 expression to inhibit the IFN-γ-induced anti-*T. gondii* response in human cell lines

**Figure 5:**
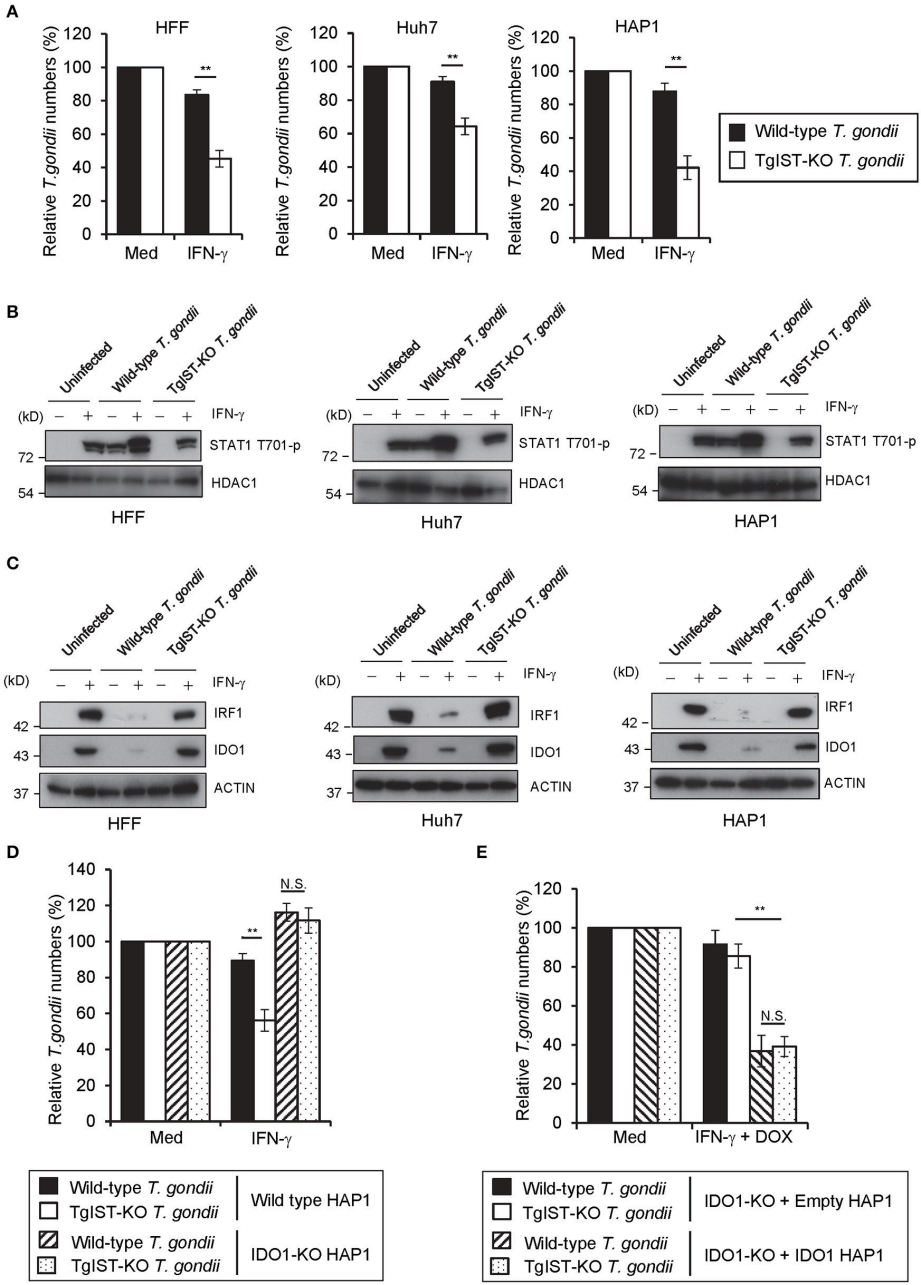
TgIST directly inhibits IDO1 mRNA induction in IFN-γ-post-treated human cells **(A)** HFFs, Huh7, or HAP1 cells were infected with WT or TgIST-KO *T. gondii* for 8 h, and subsequently treated with IFN-γ for 24 h or untreated. The parasite survival rate after 24 h post IFN-γ treatment was measured by luciferase assay. **(B,C)** HFFs, Huh7 or HAP1 cells infected with WT or TgIST-KO *T. gondii* for 8 h or uninfected, and subsequently treated with IFN-γ for 24 h or untreated. Cell lysates were detected by Western blot to detect phospho-STAT1 and HDAC1 **(B)** or IRF1, IDO1, and Actin **(C)**. **(D)** WT or IDO1-KO HAP1 cells were infected with WT or TgIST-KO *T. gondii* for 8 h, and subsequently treated with IFN-γ for 24 h or untreated. The parasite survival rate after 24 h post IFN-γ treatment was measured by luciferase assay. **(E)** IDO1-KO + Empty or IDO1-KO + IDO1 HAP1 cells were infected with WT or TgIST-KO *T. gondii* for 8 h, and subsequently treated with IFN-γ and doxycycline for 24 h or untreated. The parasite survival rate 24 h post treatment was measured by luciferase assay. Western blot image is representative of three independent experiments **(B,C)**. Indicated values are means of ± s.d. (three biological replicates per group from three independent experiments) **(A,D,E)**. **p < 0.01; N.S., not significant; (Student's *t*-test).

**Figure 6:**
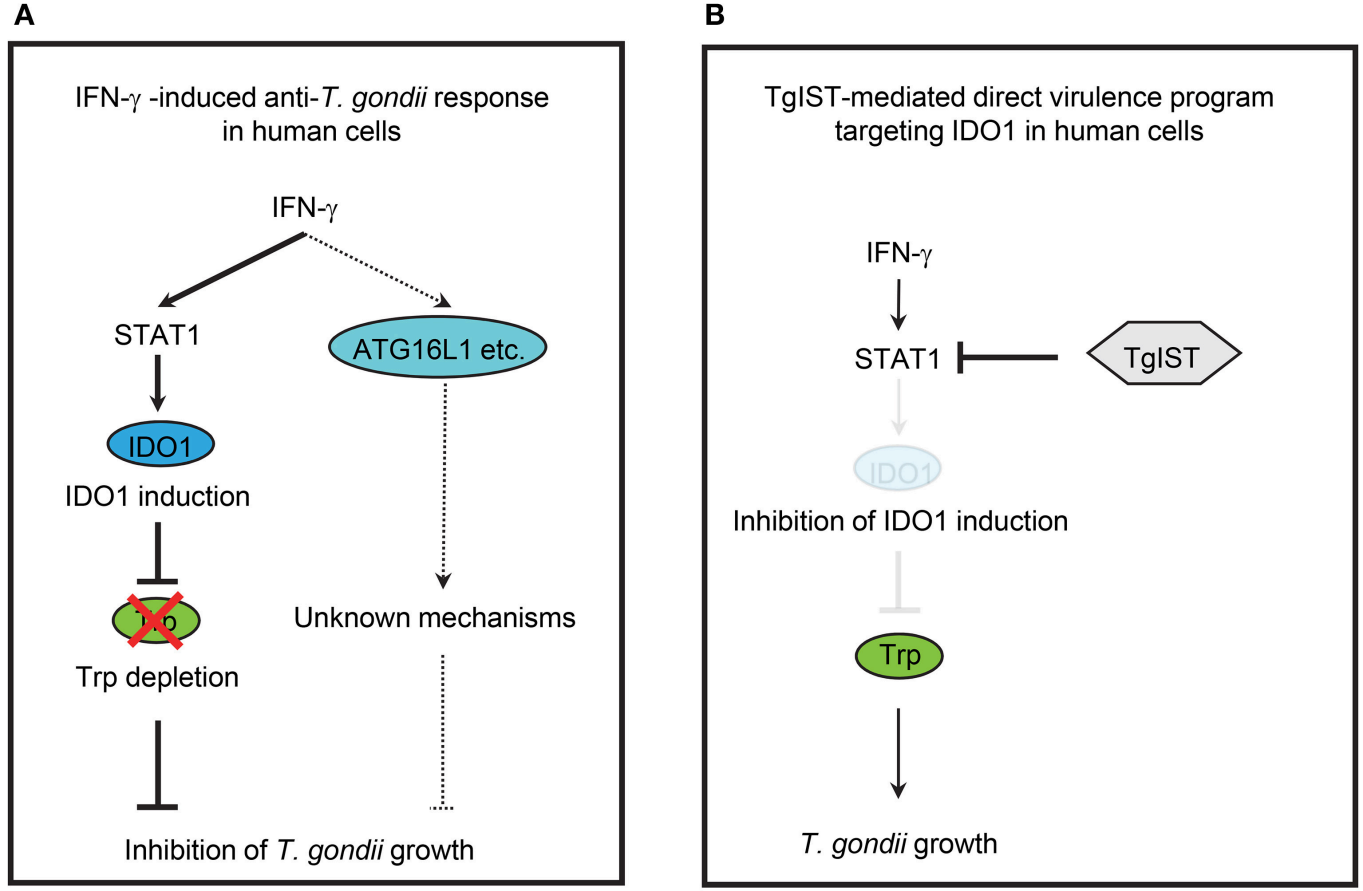
Simplified scheme of IFN-γ-induced anti-*T. gondii* host immune response and *T. gondii* virulence programs in human cells. **(A)** IFN-γ-induced anti-*T. gondii* response in human cells. IFN-γ induce the expression of IDO1, which results in depletion of L-tryptophan (Trp), leading to inhibition of *T. gondii* growth in various human cells. IFN-γ also induce ATG16L1-dependent cell—autonomous response, but the involvement of ATG16L1 in the human IFN-γ-induced anti-*T. gondii* response is cell type-specific manner. **(B)** TgIST-mediated direct virulence program targeting IDO1 in human cells. *T. gondii* secrete effector TgIST in the infected human cells, which results in the inhibition of host STAT1-dependent gene expression, leading to directly inhibition of IDO1 mRNA induction and allowing the *T. gondii* growth.

Since IDO1 plays a critical role in the IFN-γ-induced anti-*T. gondii* response in various human cells, we next explored the possible *T. gondii* virulence mechanisms targeting IDO1 in human cells. We selected TgIST, a *T. gondii* secreting effector molecule, as the candidate (47, 48), since the regulation of IDO1 expression by IFN-γ was shown to be regulated by STAT1 (52). To assess this possibility, we generated TgIST-KO type II parasites by CRISPR/Cas9 genome editing (Figures S3A,B) and asked whether TgIST deficiency affects *T. gondii* virulence in human cells (Figure 5A). As previously reported (47, 48), we also confirmed that TgIST-dependent suppression of the anti-*T. gondii* effect could be detected only when *T. gondii*-infected cells were subsequently stimulated with IFN-γ but not when IFN-γ-pre-treated cells were followed by *T. gondii* infection (Figure 5A, Figure S4A). By contrast, we observed an IFN-γ-dependent reduction in TgIST-KO parasite numbers (Figure 5A), suggesting that TgIST promotes parasite growth in the IFN-γ-post-stimulated cells. Phosphorylation of the Y701 residue of STAT1 (STAT1 Y701-p) was previously shown to be induced by *T. gondii* infection without IFN-γ stimulation and STAT1 Y701-p proteins were translocated to the nucleus (48). TgIST directly binds to STAT1 and recruits the chromatin-modifying Mi-2/NuRD complex to STAT1 transcriptional complexes. As a result, chromatin is remodeled and interferon-stimulated gene expression, including that of IRF-1, is decreased (47, 48). We tested whether *T. gondii* infection inhibits STAT1-dependent transcription in IFN-γ-post-treated HFF, Huh7, or HAP1 cells in a TgIST-dependent manner. Although phospho-STAT1 (STAT1 Y701-p) proteins were not detected in the nucleus in unstimulated cells, STAT1 Y701-p proteins were translocated to the nucleus upon IFN-γ-treatment. As previously reported (47, 48), wild-type *T. gondii* infection caused STAT1 Y701-p nuclear translocation even in unstimulated cells (Figure 5B). The subsequent IFN-γ-stimulation further induced translocation of STAT1 Y701-p to the nucleus (Figure 5B); however, STAT1 Y701-p was not active since STAT1-regulated gene products such as IRF1 and IDO1 mRNAs and proteins were not induced (Figure 5C, Figures S4B,C). By contrast, infection of TgIST-KO *T. gondii* did not result in STAT1 Y701-p nuclear translocation in unstimulated cells. Furthermore, the subsequent IFN-γ stimulation in TgIST-KO parasite-infected cells normally induced STAT1 Y701-p nuclear translocation in comparison with uninfected cells. Moreover, normal levels of IFN-γ-induced IRF1 and IDO1 expression suggested that the STAT1 activity in TgIST-KO *T. gondii*-infected cells was normal (Figure 5C, Figures S4B,C). Furthermore, IDO1-KO cells did not exhibit an IFN-γ-induced reduction in TgIST-KO parasites (Figure 5D). Conversely, growth of TgIST-intact wild-type *T. gondii* as well as TgIST-KO parasite growth was strongly inhibited by doxycycline-induced (thereby, STAT1-independent) IDO1 overexpression (Figure 5E), indicating that the pro-parasitic role of TgIST for *T. gondii* growth in IFN-γ-post-stimulated human cells is mainly due to inhibition of IDO1 but not other IFN-γ-inducible proteins (Figure 6B).

## Discussion

In the present study, we have demonstrated that IDO1 but not IDO2 plays an essential role in the IFN-γ-induced anti-*T. gondii* response in several human cell lines and primary fibroblasts. This finding is further strengthened by subsequent findings that treatment of an IDO inhibitor 1-DL-MT, consisting of 1-D-MT and 1-L-MT, reverses IFN-γ-dependent growth inhibition (32). However, IDO2 as well as IDO1 are possibly inhibited by both 1-D-MT and 1-L-MT (35, 36). Thus, whether the inhibitory effect of 1-DL-MT is on IDO1 or/and IDO2 remained unclear. Here we found that, by complete genetic deletion of IDO1 or/and IDO2 in HAP1 cells, IDO2-KO cells were able to reduce parasite numbers similarly to wild-type cells. By contrast, IDO1-KO cells as well as IDO1/IDO2-DKO cells could not control the parasite in response to IFN-γ. In addition, *T. gondii* numbers per vacuole at 24 h post infection in IDO1-KO cells were higher than in wild-type cells, whereas the infection rates were comparable. Thus, we have formally clarified that IDO1 is more important for the IFN-γ-induced inhibition of *T. gondii* growth in human cells than IDO2. Moreover, given that assessment in *T. gondii* numbers in PVs and the infection rate have been established as being indicative of the restriction of parasite replication and of parasite elimination/killing (20), IDO1 is required for *T. gondii* replication control rather than parasite elimination.

The ATG16L1-dependent cell-autonomous response mediated by IFN-γ-inducible GTPases such as IRGs and GBPs is important for the IFN-γ-mediated anti-*T. gondii* response in mice (12, 13). However, humans lack most of the IRGs (21). Moreover, human GBPs have been shown to be dispensable for the IFN-γ-inducible response (12, 24, 29). Recently, the IFN-γ-induced ubiquitination of *T. gondii* PVs has been shown to be required for the anti-*T. gondii* response in human cells such as HeLa cells and HUVECs (23, 27). Furthermore, ATG16L1 together with ATG7 are also important for the ubiquitin-mediated reduction of parasite numbers in HeLa cells (23). We have confirmed the anti-parasitic role of ATG16L1 in HeLa cells in this study. Moreover, although ATG16L1 is dispensable for the IFN-γ-induced reduction of *T. gondii* numbers in HAP1 cells, HFFs, and Huh7 cells, this autophagy protein is required in HeLa cells. The specific participation of ATG16L1 in the anti-*T. gondii* response in HeLa cells might be partly due to constitutively high levels of basal autophagy in HeLa cells (53, 54). Although the involvement of ATG16L1 in the human IFN-γ-induced anti-*T. gondii* response may be cell type-specific, complete genetic deletion of IDO1 in all human cells tested led to the impaired control of *T. gondii* growth by IFN-γ, suggesting that IDO1 is mainly used for the IFN-γ-mediated anti-*T. gondii* response in normal cells and various human cell lines. Given that ATG16L1-KO HeLa cells treated with 1-DL-MT still exhibit a modest IFN-γ-dependent reduction of *T. gondii* numbers, an ATG16L1 and IDO-independent anti-*T. gondii* response may exist in HeLa cells. It has been shown that IFN-γ stimulates cell death before parasite replication, reducing the parasite number in HFFs. In addition, IFN-γ-inducible cell death is independent of ATG5 and IDOs (29). Although we have not tested cell death in 1-DL-MT-treated ATG16L1-KO HeLa cells, it is possible that IFN-γ-dependent cell death might be responsible for the ATG16L1 and IDO-independent response.

We report a novel *T. gondii* virulence strategy in human cells, where IDO1 mRNA transcription is targeted by TgIST that directly binds to STAT1 and recruits the Mi-2/NuRD chromatin remodeling repressor complex to inhibit STAT1-dependent gene expression (47, 48). Expression of STAT1-regulated genes such as IRF1, CXCL9, CIITA, MX, GBP2, SOCS1, and ICAM1 is shown to be inhibited by TgIST in human cells (47, 48). Here we added IDO1 to the list of genes down-regulated by TgIST. Moreover, TgIST-independent (STAT1-independent) expression of IDO1 fully recovered the IFN-γ-induced growth inhibition by TgIST-sufficient parasite infection. In addition, TgIST-KO parasites can proliferate in IDO1-KO cells. Given that mRNA and protein levels of IRF1 as well as IDO1 were also inhibited in a TgIST-dependent manner, *T. gondii* may secrete TgIST to directly target STAT1 and non-specifically suppress expression of STAT1-regulated genes including IDO1 and IRF1 in the infected human cells.

In summary, we have demonstrated that IDO1 plays an important role in IFN-γ-inducible anti-*T. gondii* responses in various human cell types. Furthermore, TgIST suppresses the IFN-γ-induced anti-*T. gondii* response by directly targeting IDO1, which plays an important role in various human cells. By focusing on the difference between human and mouse immune responses, unidentified virulence mechanisms by known or unknown *T. gondii* effectors might be discovered in the future. In addition, STAT1-independent artificial induction of IDO1 could evade the TgIST-dependent virulence mechanism and offer a novel therapeutic strategy for treating human toxoplasmosis.

## Materials and methods

### Cells and parasites

All *T. gondii* strains were maintained in Vero cells in RPMI (Nacalai Tesque) supplemented with 2% heat-inactivated FBS (JRH Bioscience), 100 U/ml penicillin, and 0.1 mg/ml streptomycin (Nacalai Tesque), as previously described (55). HAP1 cells were maintained in IMDM (Nacalai Tesque) containing 10% heat-inactivated FBS, and 100 U/ml penicillin, and 0.1 mg/ml streptomycin. HFFs, Huh7 cells were maintained in RPMI (Nacalai Tesque) containing 10% heat-inactivated FBS, and 100 U/ml penicillin, and 0.1 mg/ml streptomycin. HeLa cells and MEF cells were maintained in DMEM (Nacalai Tesque) containing 10% heat-inactivated FBS, 100 U/ml penicillin, and 0.1 mg/ml streptomycin.

### Reagents

Antibodies against IDO1 (13268-1-AP), HDAC1 (10197-1-AP), and IRF1 (11335-1-AP) was obtained from Proteintech. Antibodies against Phospho-Stat1 (Tyr701) (#9167) was obtained from Cell Signaling. Antibodies against ATG16L1 (PM040) was obtained from MBL. Anti-β-actin antibody (A1978) was obtained from Sigma. Antibodies against GAP45 was described previously (56). Recombinant human and mouse IFN-γ were obtained from Peprotech. 1-Methyl-DL-tryptophan (sc-224746) was obtained from Santa Cruz Biotechnology, Inc. Aminoguanidine hydrochloride (396494) was obtained from Sigma.

### Plasmid construction for generation of human cell lines

All genomic deficient cell lines were generated with the px330 plasmid CRISPR/Cas9 system. The insert fragment of IDO1, IDO2 ATG16L1 gRNA were generated by annealing primers. All the primers used in this study are listed in Table S1. These insert fragments were inserted into the BbsI site of the cloning vector containing U6 promoter to generate gRNA expressing plasmids pIDO1_gRNA1, pIDO1_gRNA2, pIDO2_gRNA1, pIDO2_gRNA2, pATG16L1_gRNA1, and pATG16L1_gRNA2, respectively. The insert fragment was cut out by XhoI and SalI from the pIDO1_gRNA2, pIDO2_gRNA2, and pATG16L1_gRNA2 vector, and ligated into the XhoI site of the pIDO1_gRNA1, pIDO2_gRNA1, and pATG16L1_gRNA1 vector to generate plasmids pIDO1_gRNA1/2, pIDO2_gRNA1/2, and pATG16L1_gRNA1/2. The insert fragment was cut out by KpnI and MluI from pIDO1_gRNA1/2, pIDO2_gRNA1/2, and pATG16L1_gRNA1/2 vector, respectively, and ligated into the KpnI and MluI site of the pEF6-hCas9-Puro vector.

### Generation of gene-targeted human cell lines by CRISPR/Cas9 genome editing

Human cells were electroporated with the pEF6-hCas9-Puro vector containing target gRNA1/2 using NEPA21 (nepa gene). And then 24 h post-electroporation, 0.5–5 μg/ml puromycin was added for 5–10 days to select for cells with a stably integrated. Cells were plated in limiting dilution in 96-well plates to isolate single cell clones. To confirm complete target gene deficient, the IDO1, IDO2 and ATG16L1 protein expression were analyzed by Western blot. Atg16L1 KO MEF cells were described previously (12).

### Plasmid construction for generation of knockout *T. gondii* strain

Plasmid pSAG1::Cas9-U6::sgUPRT that encoding Cas9 nuclease (GFP fusion) under control of the *T. gondii* SAG1 promotor was obtained from addgene (plasmid 54467). The primer sequences are listed in Table S1. The TgIST-targeting CRISPR/Cas9 plasmid (pSAG1::Cas9-U6::sgTgIST-1 or pSAG1::Cas9-U6::sgTgIST-2) was constructed in two steps. First, the overlap PCR method was used to generate gRNA expressing plasmids. The U6 promoter from ME49 driving expression of the TgIST specific sgRNA (pgTgIST-1 or pgTgIST-2) was amplified from pSAG1::Cas9-U6::sgUPRT. For first-step PCR, primer pairs TgU6_F and TgISTgRNA1-R, TgISTgRNA1-F and TgU6_R, TgU6_F and TgISTgRNA2-R, TgISTgRNA2-F and TgU6_R were used. For second-step PCR primer pairs TgU6_F and TgU6_R were used, and cloning it into the NotI and SacI sites of the pBluescript II SK(+) (plasmid 54467). Second, the Cas9-Ds-Red monomer cassette under the SAG1 promoter was cut out from pSAG1::Cas9-U6::sgUPRT (Ds-Red monomer fusion), and ligated into the KpnI and NotI site of the pgTgIST-1 or pgTgIST-2. To generate a construct for deleting the entire coding sequence of TgIST, flanking regions of 5′ outside the sgTgIST-1 and 3′ outside the sgTgIST-2 regions were used to surround the TgIST cassette. To generate a plasmid for inserting HXGPRT into the TgIST gene, upstream regions of sgTgIST-1 (894-bp) and downstream regions of sgTgIST-2 (670-bp) were amplified from ME49 genomic DNA by using primers TgIST targeting 5′_F and TgIST targeting 5′_R or TgIST targeting 3′_F and TgIST targeting 3′_R. These two fragments were ligated into the KpnI and XhoI or BamHI and NotI sites of pHXGPRT vector.

### Generation of TgIST-KO *T. gondii* by CRISPR/Cas9 genome editing

Prugniaud (Pru) *T. gondii*-expressing luciferase were filtered, washed and resuspended in Cytomix (10 mM KPO_4_, 120 mM KCl, 0.15 mM CaCl_2_, 5 mM MgCl_2_, 25 mM HEPES, 2 mM EDTA). Parasites were mixed with 50 μg of sgTgIST-1 and sgTgIST-2 CRISPR plasmid along with 40 μg of the targeting vector linearized by Kpnl and SacI, and supplemented with 2 mM ATP, 5 mM GSH. Parasites were electroporated by GENE PULSER II (Bio-Rad Laboratories). Selection by growth for 14 days in 25 μg/ml mycophenolic acid (Sigma) and 25 μg/ml xanthine (Wako) were used to obtain stably resistant clone. And then parasites were plated in limiting dilution in 96-well plates to isolate single clones. To confirm the disruption of the gene encoding TgIST, we analyzed messenger RNA of TgIST from WT and TgIST-KO parasites by quantitative RT-PCR. In addition, we observed comparable *in vitro* growth and *in vivo* virulence to each other and to the parental line.

### Quantitative RT-PCR

Total RNA was extracted, and cDNA was synthesized using Verso Reverse transcription (Thermo Fisher Scientic). Quantitative RT-PCR was performed with a CFX connect real-time PCR system (Bio-Rad Laboratories) using the Go-Taq Real-Time PCR system (Promega). The values were normalized to the amount of beta actin (β-actin) for human cells or tubulin for *T. gondii* in each sample. The primer sequences are listed in Table S1.

### Western blot analysis

Cells were lysed in a lysis buffer (0.5% Nonidet P-40, 150 mM NaCl, and 20 mM Tris-HCl, pH 7.5) containing a protease inhibitor cocktail (Roche) and phosphatase inhibitor cocktail (Nacalai Tesque). The cell lysates were separated by SDS-PAGE and transferred to polyvinylidene uoride membranes and subjected to Western blot analysis using the indicated antibodies as described previously (57).

### Luciferase assay

For the experiment using IFN-γ pre-stimulated cells, HFFs (6 × 10^5^), HAP1 (2 × 10^6^), HeLa (8 × 10^5^), or Huh7 (6 × 10^5^) cells were untreated or treated with 10 ng/ml IFN-γ for 24 h, and subsequently infected with the luciferase-expressing *T. gondii* (MOI = 0.5) for 24 h. For the experiment using IFN-γ post-stimulation cells, HFFs (6 × 10^5^), HAP1 (2 × 10^6^), HeLa (8 × 10^5^), or Huh7 (6 × 10^5^) cells were infected with the luciferase-expressing *T. gondii* (MOI = 0.5) for 8 h, and subsequently untreated or treated with 10 ng/ml IFN-γ for 24 h. To measure the number of *T. gondii*, all infected cells were collected and lysed by 100 μl of lysis buffer (Promega) and sonicated. After centrifugation at 20,000 × g at 4°C, the luciferase activity of the supernatant was measured using the Dual Luciferase Reporter Assay System (Promega) and GLOMAX 20/20 luminometer (Promega). The percentages of the activities in cytokines stimulated cells over those in unstimulated cells were shown as “Relative *T. gondii* numbers” in figures.

### Immunofluorescence assays

HFFs, Huh7, or HeLa cells were cultured on glass coverslips, and infected with *T. gondii* (MOI = 2) for the indicated time, and fixed in PBS containing 3.7% paraformaldehyde for 10 min at room temperature. Cells were permeabilized with PBS containing 0.002% Digitonin for 5 min and then blocked with 8% FBS in PBS for 1 h at room temperature. And then, cells were incubated with the indicated primary antibodies for 1 h at 37°C, followed by incubation with Alexa 488-, Alexa 594-, or Alexa 647-conjugated secondary antibodies (Molecular Probes) and DAPI for 1 h at 37°C in the dark. Finally, coverslips were mounted onto glass slides with PermaFluor (Thermo Scientific) and analyzed using confocal laser microscopy (FV1200 IX-83, Olympus).

### IDO activity assay

The enzymatic IDO activity was evaluated by the calculation of the kynurenine concentration in the cell culture supernatant as previously described (58). Cells were cultured in 12-well plates and untreated or treated with 10 ng/ml IFN-γ for 24 h. The concentration of kynurenine in culture supernatant was measured using Ehrlich reagent method (59). Seventy microliters of culture supernatant was mixed with 35 μl of 30% trichloroacetic acid, and centrifuged at 8,000 x g for 5 min. Then 75 μl of the supernatant was added to an equal volume of Ehrlich reagent (0.8% p-dimethylaminobenzaldehyde in acetic acid) in a 96-well plate, and the absorbance was read at 490 nm. The values were compared with a standard curve with defined concentrations of kynurenine (Sigma Aldrich).

### Statistical analysis

All statistical analyses were performed using Excel (Microsoft). All the experimental points and n values represent an average of each three biological replicates (three independent experiments). The statistical significance of differences in mean values was analyzed by using an unpaired two-tailed Student's *t*-test. *P*-values less than 0.05 were considered to be statistically significant.

## Author contributions

HB, NS, ST, YN, and MY designed the experiments. AP, JM, ST, and MS prepared the materials. HB, NS, JL, Z-RL, and MY analyzed the data. HB, NS, DL, Z-RL, and MY wrote the paper. All authors listed contributed to revised work and approved the final manuscript.

### Conflict of interest statement

The authors declare that the research was conducted in the absence of any commercial or financial relationships that could be construed as a potential conflict of interest.
