# Supplementary material for: Toxoplasma Effector TgIST Targets Host IDO1 to Antagonize the IFN-γ-Induced Anti-parasitic Response in Human Cells
Source: Front Immunol. 2018 Sep 19;9:2073. doi: 10.3389/fimmu.2018.02073 (PMC6156249; doi:10.3389/fimmu.2018.02073)
Supplement: Supplementary file 1 [file Data_Sheet_1.pdf]

Supplementary Figure and Table

Toxoplasma effector TgIST targets host IDO1 to antagonize the IFN-γ-induced anti-parasitic response in human cells

Hironori Bando[1,2†], Naoya Sakaguchi [1†], Youngae Lee[1,2], Ariel Pradipta[1], Ji Su Ma[1,2], Shun Tanaka[1,2], Dehua Lai [3], Jianfa Liu[4], Zhao-Rong Lun[3], Yoshifumi Nishikawa[5], Miwa Sasai [1,2] and Masahiro Yamamoto[1,2*]

[1] Department of Immunoparasitology, Research Institute for Microbial Diseases, Osaka University, Osaka, Japan,

[2] Laboratory of Immunoparasitology, WPI Immunology Frontier Research Center, Osaka University, Osaka, Japan,

[3] State Key Laboratory of Biocontrol, Center for Parasitic Organisms, School of Life Sciences, Guangzhou, China,

[4] Department of Pathology and Pathogenic Biology, Medical College of Ningbo University, Ningbo, China,

[5] National Research Center for ProtozoanDiseases, Obihiro University of Agriculture and Veterinary Medicine, Obihiro, Japan

[†]These authors have contributed equally to this work

* Correspondence should be addressed to:

Masahiro Yamamoto

Department of Immunoparasitology, Research Institute for Microbial Diseases, Osaka University, 3-1, Yamadaoka, Suita, Osaka, Japan, 565-0871

Tel: +81-6-6879-8333, Fax: +81-6-6879-8332

Figure S1_Bando et al.

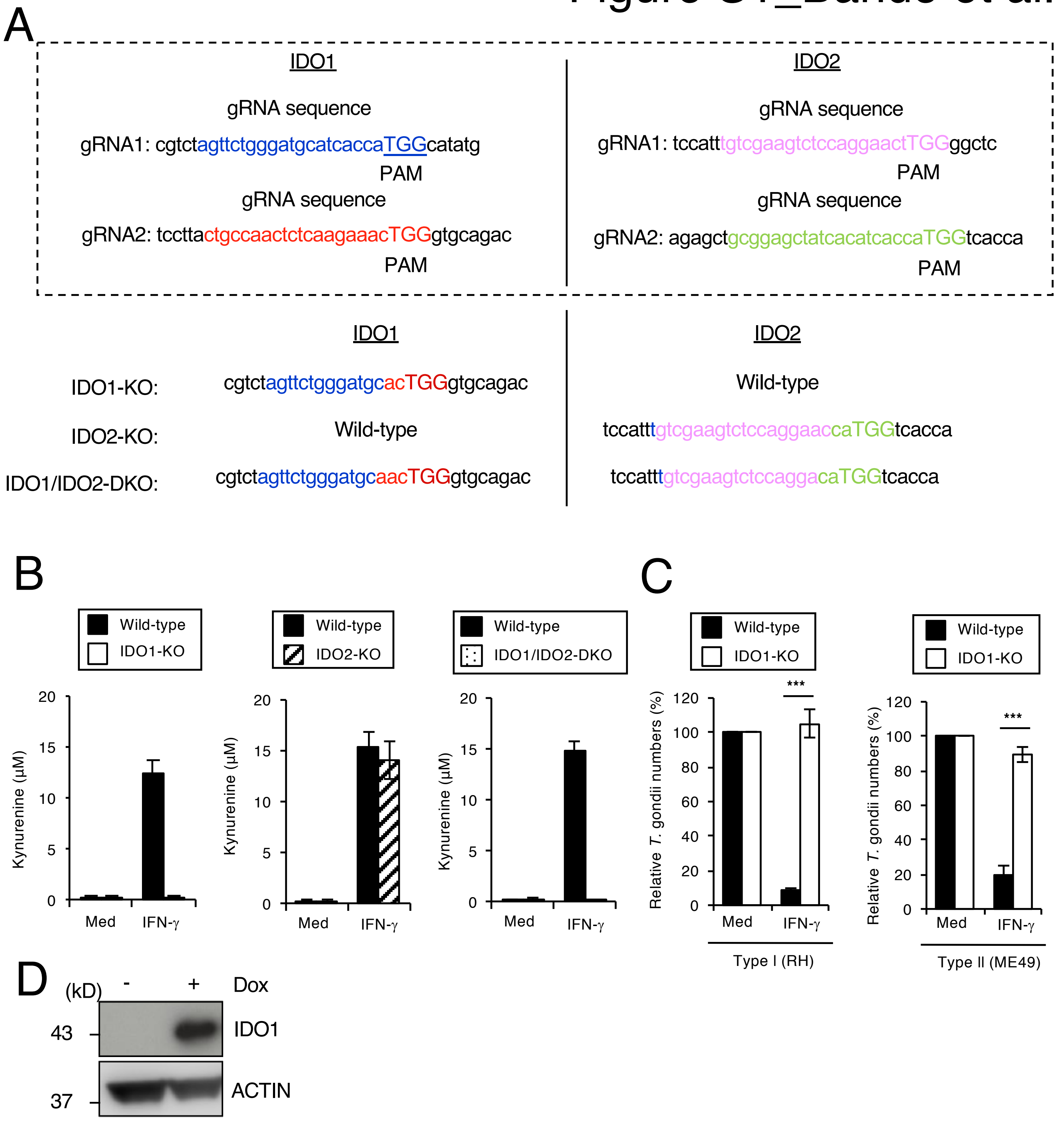

FIGURE S1. Generation of IDO1- and/or IDO2-deficient HAP1 cells by CRISPR/Cas9 genome editing. (**A**) Generated IDO1- and/or IDO2-deficient HAP1 cells were confirmed by sequence analysis. (**B**) IDO1- and/or IDO2-deficient HAP1 cells were untreated or treated with IFN-γ for 24 hours, and then the concentration of kynurenine in the cell culture supernatant was measured. **(C)** WT or IDO1-KO HAP1 cells were untreated or treated with IFN-γ for 24 hours, and then infected with Type I or Type II *T. gondii*. The parasite survival rate after 24 hours post infection was measured by luciferase assay. **(D)** IDO1-KO + IDO1 HAP1 cells were untreated or treated with IFN-γ and doxycycline for 24 hours. Cell lysates were detected by Western blot. Western blot image is representative of three independent experiments (**D**). Indicated values are means of ± s.d. (three biological replicates per group from three independent experiments) (**B, C**). *** $p < 0.001$ (Student's t-test).

Figure S2_Bando et al.

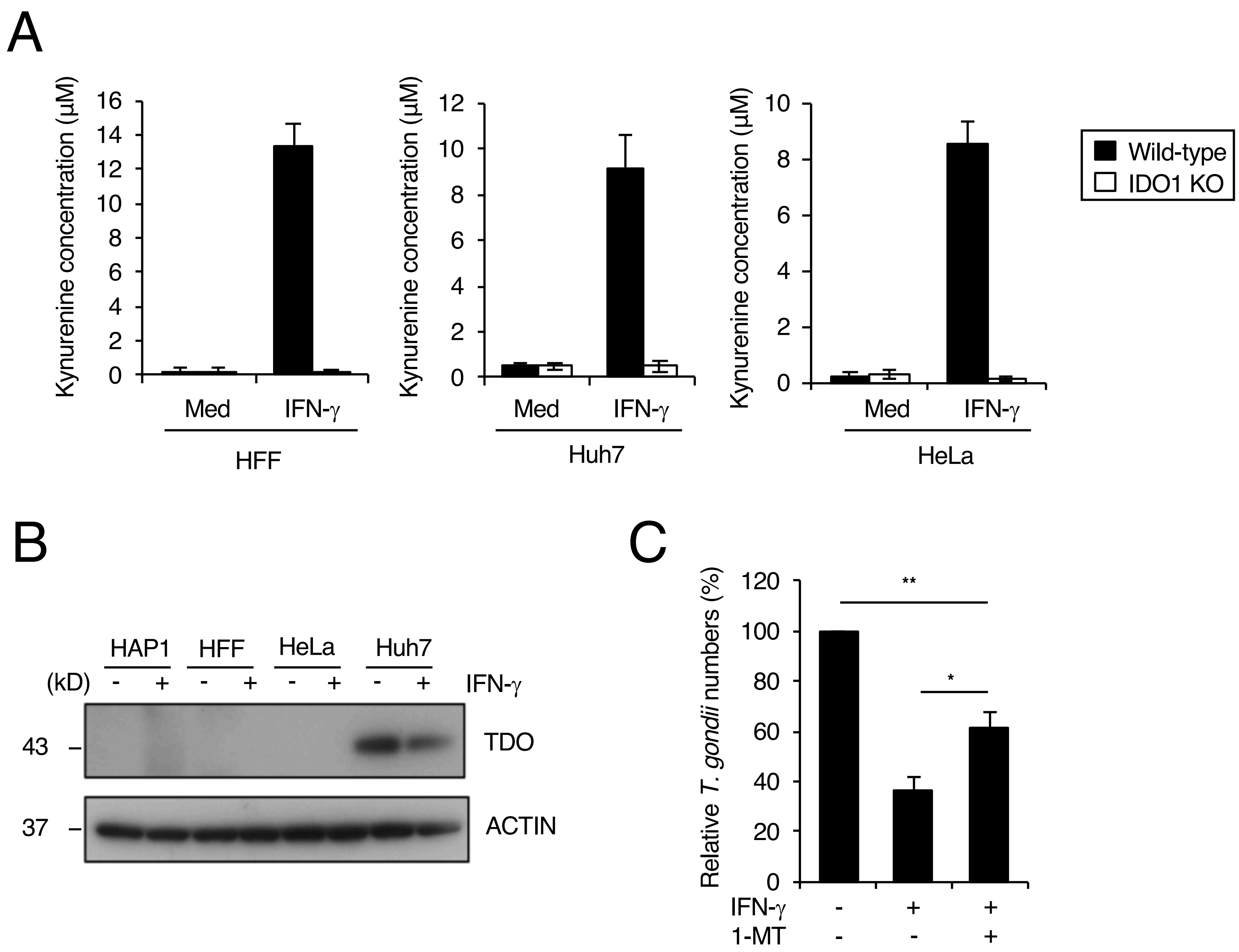

FIGURE S2. **Generation of IDO1-deficient HFFs, Huh7 cells and HeLa cells by CRISPR/Cas9 genome editing.**

(**A**) IDO1-deficient HFFs, Huh7 cells and HeLa cells were untreated or treated with IFN-γ for 24 hours, and then the concentration of kynurenine in the cell culture supernatant was measured. **(B)** HFFs, HAP1, HeLa and Huh7 cells were untreated or treated with IFN-γ for 24 hours. Cell lysates were detected by Western blot. (**C**) ATG16L1-KO HeLa cells were untreated or treated with IFN-γ and/or 1-DL-MT for 24 hours, and then infected with *T. gondii*. The parasite survival rate after 24 hours post infection was measured by luciferase assay. Western blot image is representative of three independent experiments (**B**). Indicated values are means of ± s.d. (three biological replicates per group from three independent experiments) (**A, C**). * $p < 0.05$, ** $p < 0.01$; (Student's t-test).

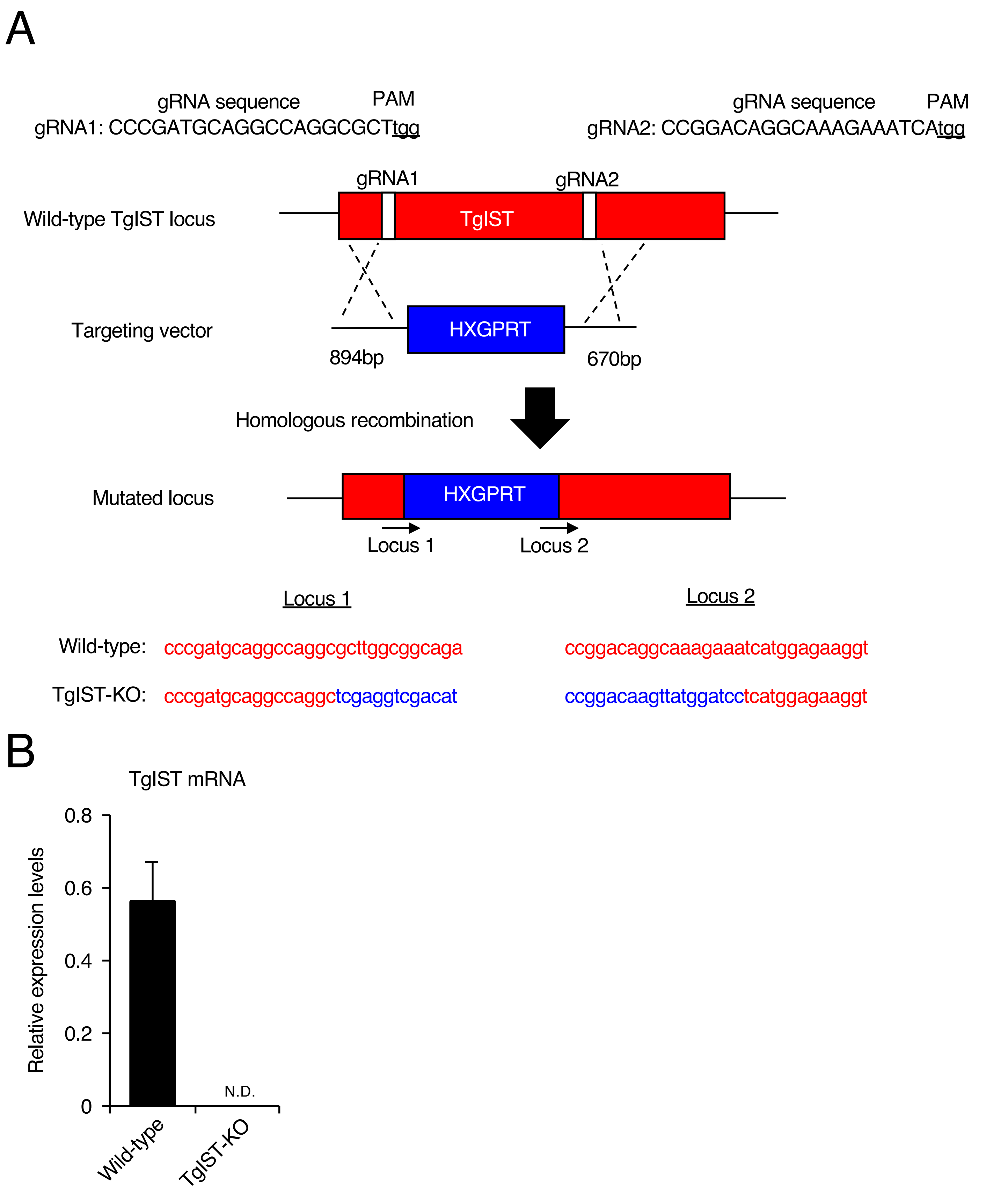

FIGURE S3. Generation of TgIST-deficient *T. gondii* by CRISPR/Cas9 genome editing. (**A**) Schematic of CRISPR/Cas9-mediated disruption of TgIST by insertion of HXGPRT. Transfection of sg TgIST-1 and sg TgIST-2 together with the HXGPRT amplicon shown was used to disrupt the TgIST coding region. The white bar indicates the sg TgIST-1 and sg TgIST-2 target region. (**B**) Quantitative RT-PCR analysis of TgIST mRNA level in WT or TgIST-KO *T. gondii* was performed, and the data was normalized to the mRNA expression levels of tubulin in each samples.

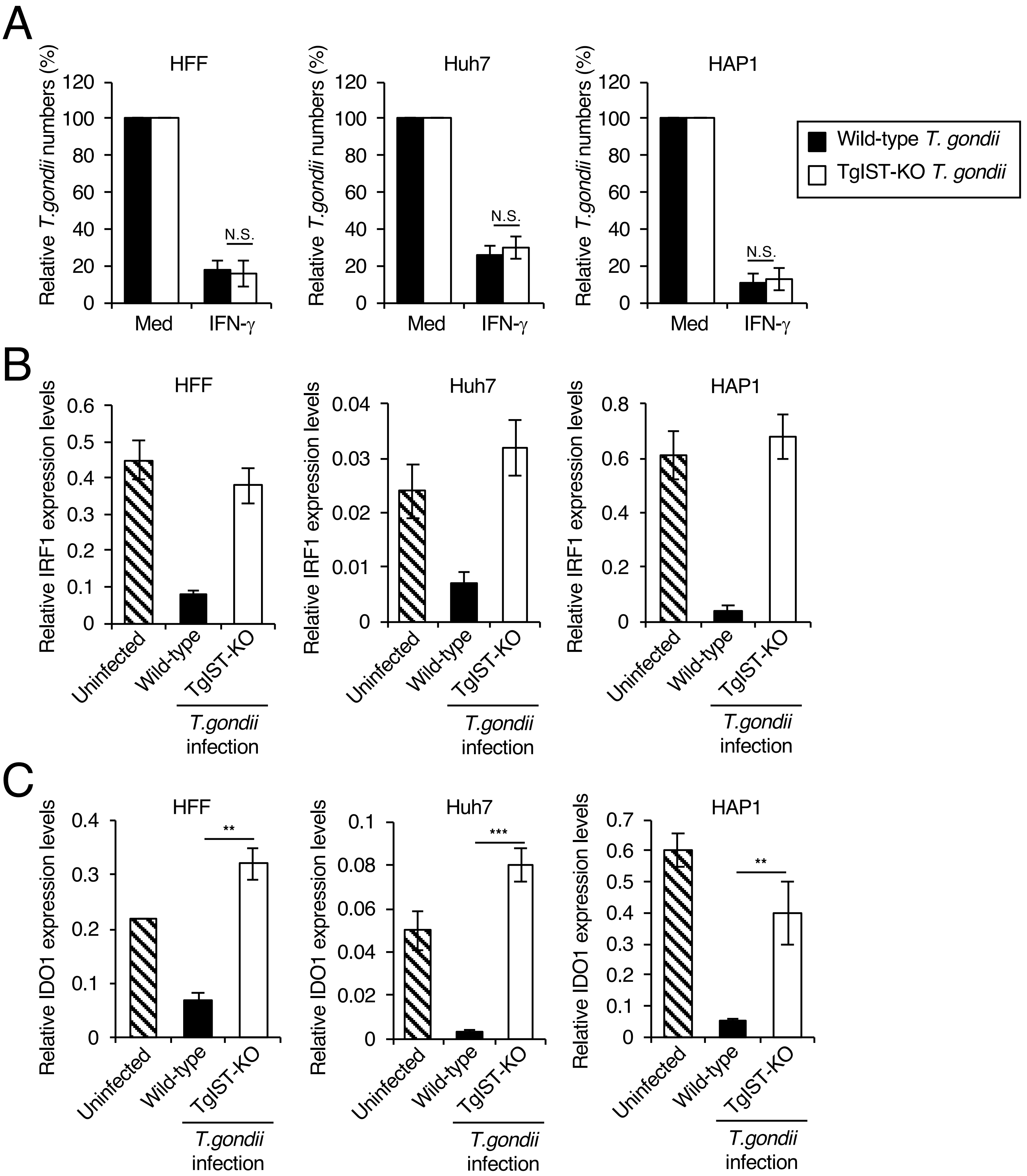

FIGURE S4. TgIST targets IDO1 and IRF1 to suppress IFN-γ-induced parasite reduction.
(A) HFFs, Huh7 or HAP1 cells were untreated or pre-treated with IFN-γ for 24 hours, and then infected with WT or TgIST-KO *T. gondii*. The parasite survival rate after 24 hours post infection was measured by luciferase assay. **(B and C)** Quantitative RT-PCR analysis of IRF1 (B) or IDO1 (C) mRNA level in HFFs, Huh7 or HeLa cells that were untreated or treated with IFN-γ for 24 hours, and infected with or without WT or TgIST-KO *T. gondii* was performed. Indicated values are means of ± s.d. (three biological replicates per group from three independent experiments) (**A, B, C**). ** $p < 0.01$, *** $p < 0.001$; N.S., not significant; (Student's t-test).

TABLE S1.

Primer name	enzyme	sequence	Resulting plasmids and descriptions
IDO1_gRNA1_F	—	5'-CACCGagttctgggatgcatcacca-3'	IDO1 gRNA1
IDO1_gRNA1_R	—	5'-AAACtggtgatgcatcccagaactC-3'	IDO1 gRNA1
IDO1_gRNA2_F	—	5'-CACCGctgccaactctccaagaaac-3	IDO1 gRNA2
IDO1_gRNA2_R	—	5'-AAACgtttcttggagagttggcagC-3'	IDO1 gRNA2
IDO2_gRNA1_F	—	5'-CACCGtgtcgaagtctccaggaact-3'	IDO2 gRNA1
IDO2_gRNA1_R	—	5'-AAACagttcctggagacttcgacaC-3'	IDO2 gRNA1
IDO2_gRNA2_F	—	5'-CACCGgcggagctatcacatcacca-3'	IDO2 gRNA2
IDO2_gRNA2_R	—	5'-AAACtggtgatgtgatagctccgcC-3'	IDO2 gRNA2
iNOS_gRNA1_F	—	5'- CACCGccttgtgcttgaggtggcca -3'	iNOS gRNA1
iNOS _gRNA1_R	—	5'- AAACtggccacctcaagcacaaggC -3'	iNOS gRNA1
iNOS_gRNA2_F	—	5'- CACCGcaccaacagccccacattcc -3'	iNOS gRNA2
iNOS _gRNA2_R	—	5'- AAACggaatgtggggctgttggtgC-3'	iNOS gRNA2
ATG16L1_gRNA1_F	—	5'-CACCGtctcggagcaactgaggcgc-3'	ATG16L1 gRNA1
ATG16 L1_gRNA1_R	—	5'-AAACgcgcctcagttgctccgagaC-3'	ATG16L1 gRNA1
ATG16 L1_gRNA2_F	—	5'-CACCGggagctggtcaccagatgga-3'	ATG16L1 gRNA2
ATG16 L1_gRNA2_R	—	5'-AAACtccatctggtgaccagctccC-3'	ATG16L1 gRNA2
DsRed-Monomer_F	BamHI	5'-GGATCCGACAACACCGAGGACGTCATCAAG-3'	pSAG1::Cas9-U6::sgUPRT (Ds-Red monomer fusion)
DsRed-Monomer_R	PacI	5'-TTAATTAACTAGGACTGGGAGCCGGAGTGGCGGGCCTCGGCGTG-	pSAG1::Cas9-U6::sgUPRT (Ds-Red monomer fusion)
TgU6_F	NotI	5'-GCGGCCGCCACCGCGGTGGAGCTCAAGTAAGCAGAAGCACGCTG	pgGRA15-1, pgGRA15-2, pgTgIST-1, pgTgIST-2
TgU6_R	SacI	5'-GAGCTCAAAAAAGCACCGACTCGGT-3'	pgGRA15-1, pgGRA15-2, pgTgIST-1, pgTgIST-2
TgISTgRNA1-F	—	5'-CCCGATGCAGGCCAGGCGCTGTTTTAGAGCTAGAAATAGCAAGT-3	pgTgIST-1
TgISTgRNA1-R	—	5'-AGCGCCTGGCCTGCATCGGGAACTTGACATCCCCATTTACCAGA-3	pgTgIST-1
TgISTgRNA2-F	—	5'-CCGGACAGGCAAAGAAATCAGTTTTAGAGCTAGAAATAGCAAGT-3'	pgTgIST-2
TgISTgRNA2-R	—	5'-TGATTTCTTTGCCTGTCCGGAACTTGACATCCCCATTTACCAGA-3'	pgTgIST-2
TgIST targeting 5'_F	KpnI	5'-GGTACCCTTCGAGGCTGTCCCCGCC-3'	pHXGPRT-TgIST-5'
TgIST targeting 5'_R	XhoI	5'-CTCGAGGCCTGGCCTGCATCGGGGC-3'	pHXGPRT-TgIST-5'
TgIST targeting 3'_F	BamHI	5'-GGATCCTCATGGAGAAGGTGAAAAA-3'	pHXGPRT-TgIST-3'
TgIST targeting 3'_R	NotI	5'-GCGGCCGCGTCCGCGGTCGGACGAG-3'	pHXGPRT-TgIST-3'
hIDO1_F	—	5'-AGTGTTTCACCAAATCCACGATC-3'	Quantitative RT-PCR
hIDO1_R	—	5'-AAGCACTGAAAGACGCTGCTTTG-3'	Quantitative RT-PCR
hIDO2_F	—	5'-GACTGTCTATTCAGGACATCACC-3'	Quantitative RT-PCR
hIDO2_R	—	5'-GGAAGGAGGCATGTAATCCCTCA-3'	Quantitative RT-PCR
hiNOS_F	—	5'-CACAGAGATCCACCTGACTGTGG-3'	Quantitative RT-PCR
hIRF1_F	—	5'-CATGAGACCCTGGCTAGAGATGC-3'	Quantitative RT-PCR
hIRF1_R	—	5'-ATGCTTGGCAGCATGCTTCCATGG-3'	Quantitative RT-PCR
hiNOS_R	—	5'-AAGCCGCTGGCATTCCGCACAAA-3'	Quantitative RT-PCR
hβ-actin_F	—	5'-CATGTACGTTGCTATCCAGGC-3'	Quantitative RT-PCR
hβ-actin_R	—	5'-CTCCTTAATGTCACGCACGAT-3'	Quantitative RT-PCR
TgIST_F	—	5'-GCAGAGGGTGGAAGTGAATCAGAAG-3'	Quantitative RT-PCR
TgIST_R	—	5'-CTGGTTCGGAACCACCTGGAGTGGA-3'	Quantitative RT-PCR
Tgtublin_F	—	5'-TGTCTGTCGCTGAGATCACCAACT-3'	Quantitative RT-PCR
Tgtublin_R	—	5'-TCTCTTGGTCTTGATGGTCGCAAC-3'	Quantitative RT-PCR
